# Lack of Correlation between Stem-Cell Proliferation and Radiation- or Smoking-Associated Cancer Risk

**DOI:** 10.1371/journal.pone.0150335

**Published:** 2016-03-31

**Authors:** Mark P. Little, Jolyon H. Hendry, Jerome S. Puskin

**Affiliations:** 1 Radiation Epidemiology Branch, Division of Cancer Epidemiology and Genetics, National Cancer Institute, National Institutes of Health (NIH), Department of Health and Human Services (DHHS), Rockville, Maryland, United States of America; 2 Christie Medical Physics and Engineering, Christie Hospital and University of Manchester, Manchester, United Kingdom; 3 Radiation Protection Division, United States Environmental Protection Agency, Washington, DC, United States of America; Aichi Cancer Center Research Institute, JAPAN

## Abstract

**Background:**

A recent paper by Tomasetti and Vogelstein (*Science* 2015 **347** 78–81) suggested that the variation in natural cancer risk was largely explained by the total number of stem-cell divisions, and that most cancers arose by chance. They proposed an extra-risk score as way of distinguishing the effects of the stochastic, replicative component of cancer risk from other causative factors, specifically those due to the external environment and inherited mutations.

**Objectives:**

We tested the hypothesis raised by Tomasetti and Vogelstein by assessing the degree of correlation of stem cell divisions and their extra-risk score with radiation- and tobacco-associated cancer risk.

**Methods:**

We fitted a variety of linear and log-linear models to data on stem cell divisions per year and cumulative stem cell divisions over lifetime and natural cancer risk, some taken from the paper of Tomasetti and Vogelstein, augmented using current US lifetime cancer risk data, and also radiation- and tobacco-associated cancer risk.

**Results:**

The data assembled by Tomasetti and Vogelstein, as augmented here, are inconsistent with the power-of-age relationship commonly observed for cancer incidence and the predictions of a multistage carcinogenesis model, if one makes the strong assumption of homogeneity of numbers of driver mutations across cancer sites. Analysis of the extra-risk score and various other measures (number of stem cell divisions per year, cumulative number of stem cell divisions over life) considered by Tomasetti and Vogelstein suggests that these are poorly predictive of currently available estimates of radiation- or smoking-associated cancer risk–for only one out of 37 measures or logarithmic transformations thereof is there a statistically significant correlation (*p*<0.05) with radiation- or smoking-associated risk.

**Conclusions:**

The data used by Tomasetti and Vogelstein are in conflict with predictions of a multistage model of carcinogenesis, under the assumption of homogeneity of numbers of driver mutations across most cancer sites. Their hypothesis that if the extra-risk score for a tissue type is high then one would expect that environmental factors would play a relatively more important role in that cancer’s risk is in conflict with the lack of correlation between the extra-risk score and other stem-cell proliferation indices and radiation- or smoking-related cancer risk.

## Introduction

Stem cells are a type of cell in each tissue that are responsible for maintaining tissue homeostasis. There is increasing attention devoted to stem cells and their role in the carcinogenic process [1]. A recent paper of Tomasetti and Vogelstein [2] aroused considerable interest, and suggested that “the lifetime risk of cancers of many different types [was] strongly correlated … with the total number of divisions of the normal self-renewing [stem] cells”. Tomasetti and Vogelstein suggested a causal interpretation of their findings, stating that “the incorporation of a replicative component as a … quantitative determinant of cancer risk forces rethinking of our notions of cancer causation. The contribution of the classic determinants (external environment and heredity) to [replicative, cell-division associated] tumors is minimal. Even for [deterministic, non-replicative] tumors, however, replicative [cell-division associated] effects are essential” and that “our analysis shows that stochastic effects associated with DNA replication contribute in a substantial way to human cancer incidence in the United States” [2].

Tomasetti and Vogelstein [2] analyzed data on 31 cancer types, correlating natural cancer risk for the particular tissue with its total number of stem-cell divisions. They suggested that the high degree of correlation (*ρ* = 0.804, 95% CI 0.63, 0.90), and the relatively high R^2^ (0.646, 95% CI 0.395, 0.813) implied that a high proportion, “65% … of the differences in cancer risk among various tissues can be explained by the total number of stem cell divisions in those tissues”. Therefore they attributed a large part of the variation in cancer risk to chance or “bad luck”, specifically the stochastic effects of DNA replication-induced mutations. Tomasetti and Vogelstein [2] defined an extra-risk score (ERS) for the purpose of distinguishing “the effects of this stochastic, replicative component from other causative factors—that is, those due to the external environment and inherited mutations”.

One of the more common patterns in the age-incidence curves for epithelial cancers is that the incidence rate varies approximately as *C*[age]^*β*^ for some constants *C* and *β*. For most epithelial cancers in adulthood, the exponent *β* of age seems to lie between 4 and 6 [3]. The so-called multistage model of carcinogenesis of Armitage and Doll [4] was developed as a way of accounting for this approximately log-log variation of cancer incidence with age. Much use has been made of the Armitage-Doll multistage model as a framework for understanding the time course of carcinogenesis, particularly for modeling effects of various types of occupational and environmental exposures, and the interaction of different carcinogens [5–9]. The Armitage-Doll model has also been used to determine the number of driver-gene mutations associated with two types of cancer [10]. The Armitage-Doll model [4] supposes that a malignant neoplasm arises from a normal cell as a result of *k* irreversible heritable changes (so called “driver mutations”). At a rate of *λ*_*i*_(*t*) per cell per unit time, at age *t* the cells in the compartment which have accumulated *i*−1 heritable driver mutations acquire one more. If the expected number of cells in the compartment which have accumulated *i* heritable driver mutational changes is given by *N*_*i*_(*t*) (with *N*_0_ stem cells per tissue), then if the driver mutation rates are assumed constant (*λ*_*m*_(*t*) ≡ *λ*_*m*_) the cancer incidence rate at age *t* is approximately given [11] by:

$$\frac{dN_{k}(t)}{dt}\approx N_{0}\lambda_{1}\ldots \lambda_{k}t^{k-1}/(k-1)!$$ (1)

This suggests that to account for the observed variation in cancer incidence and mortality rates of *C*[age]^*β*^ with *β* between 4 and 6 [3], the number of driver mutation stages *k* should lie between 5 and 7.

This model can be easily generalized to take account of intermediate compartment growth rates in the *k* compartments, as has been done by Tomasetti *et al*. [10], based on approximations of Durrett and Moseley [12]. Assuming that the growth rate induced by the *i*th driver mutation is *g*_*i*_ then the cancer risk becomes:

$$\frac{dN_{k}(t)}{dt}\approx N_{0}\lambda_{1}\lambda_{2}{}^{g_{1}/g_{1}}\lambda_{3}{}^{g_{1}/g_{2}}\ldots \lambda_{k}{}^{g_{1}/g_{k-1}}t^{k-1}/(k-1)!$$ (2)

Expression (2) implies that the lifetime cancer risk (corrected for mortality from other causes), *CR*, is approximately:

$$CR\approx \int_{0}^{L}\frac{dN_{k}(t)}{dt}dt=N_{0}\lambda_{1}\lambda_{2}{}^{g_{1}/g_{1}}\lambda_{3}{}^{g_{1}/g_{2}}\ldots \lambda_{k}{}^{g_{1}/g_{k-1}}L^{k}/k!$$ (3)

where *L* is the expected lifetime. If all mutation rates are equal (*λ*_*m*_ ≡ *λ*) this (approximately) reduces to:

$$CR\approx N_{0}L^{k}\lambda^{1+\sum_{i=1}^{k-1}g_{1}/g_{i}}/k!$$ (4)

Since the expected number of stem-cell divisions over life, *D*, is given by:

$$D=N_{0}L\lambda$$ (5)

this implies that:

$$\text{ln}[CR]\approx F((g_{i}),k)\text{ln}[D/N_{0}]+\left[k-F((g_{i}),k)\right]\text{ln}[L]+\text{ln}[N_{0}]-\text{ln}[k!]$$ (6)

where:

$$F((g_{i}),k)=1+\left(\sum_{i=1}^{k-1}g_{1}/g_{i}\right)$$ (7)

i.e. cancer rate should be proportional to the *F*((*g*_*i*_),*k*)th power of the expected number of cell divisions per stem cell, *D* / *N*_0_, and a linear function of the number of stem cells, *N*_0_. It is also proportional to the [*k*−*F*((*g*_*i*_),*k*)]th power of the expected lifetime, *L*, but as [*k*−*F*((*g*_*i*_),*k*)] is assumed to be constant across tissues (an assumption discussed and analyzed below), as is *L*, this term together with the final −ln[*k*!] term are constant. As pointed out by Tomasetti *et al*. [10], there is little information on the growth rates *g*_*i*_. Following Tomasetti *et al*. [10] we shall therefore assume that the fitness advantage added by each successive driver mutation is constant, resulting in *g*_1_ / *g*_*k*_ = 1 / *k*, so that:

$$F((g_{i}),k)=1+\left(\sum_{i=1}^{k-1}1/i\right)\approx 1+\text{ln}[k]$$ (8)

The adequacy of approximation (8) can be gauged by the fact that with *k* = 5 the left hand side of the expression can be computed to be 3.08, compared with the right hand side of 1 + ln[5] = 2.61, and with *k* = 7 the left hand side of expression (8) can be computed to be 3.45, compared with the right hand side 1 + ln[7] = 2.95. However, we do not use approximation (8) further here. The only property we shall need of *F*((*g*_*i*_),*k*), which follows immediately from inspection of expressions (7) and (8), is that it be a monotonic increasing function of *k*.

We suggest a number of problems with the interpretation that Tomasetti and Vogelstein [2] provide. These comprise:

1. considerations arising from the standard multistage carcinogenesis model of Armitage and Doll [4] discussed above; and
2. analysis of radiation- or smoking-associated cancer risk in relation to the ERS and various other measures considered by Tomasetti and Vogelstein [2].

All analysis is based on the data presented by Tomasetti and Vogelstein [2], and is given in Tables 1 and 2 and S2 Text. Analysis of this data in the light of consideration (a) is given in Table 3, and analysis in the light of consideration (b) in Tables 4–6.

**Table 1:** Summary data on radiation-associated cancer risk and various tissue parameters, reproduced in part from Tomasetti & Vogelstein [2] and UNSCEAR [unless otherwise indicated taken from Table 70 in Annex A of [14]]

Cancer type	US lifetime natural cancer incidence [13]	Number of stem cells in tissue of origin	Numbers of divisions of each stem cell per year	Numbers of divisions of each stem cell over life	Number of divisions of all stem cell over lifetime	Extra Risk Score (ERS)	Radiation exposure-induced cancer incidence risk (percent per Sv^a^ incidence risk (evaluated at 1 Sv^a^ for current Japanese population [14])			
							Excess relative risk (ERR) model	Excess absolute risk (EAR) model	BEIR VII [19] weighted ERR/EAR model	ICRP [18] weighted ERR/EAR model
Chronic lymphocytic leukemia	0.006	1.35 x 10^8^	12	960	1.30 x 10^11^	-24.69	0	0	0	0
All leukemia	0.014	1.35 x 10^8^	12	960	1.30 x 10^11^	-20.60	0.69^b^	0.86^b^	0.74^b^	0.86^b^
Basal cell carcinoma	0.3^c^	5.82 x 10^9^	7.6	608	3.55 x 10^12^	-6.56	0.23^d^	0.55^d^	0.33^d^	0.39^d^
Colon cancer	0.045	2.00 x 10^8^	73	5840	1.17 x 10^12^	-16.25	1.3	1.36	1.32	1.33
Esophageal cancer	0.005	8.64 x 10^5^	17.4	1390	1.20 x 10^9^	-20.89	0.41	0.05	0.30	0.23
Brain and CNS cancer	0.006	1.35 x 10^8^	0.025	2	2.70 x 10	-18.73	0.16	0.17	0.16	0.17
Trachea, bronchus and lung cancer	0.066	1.22 x 10^9^	0.07	5.6	9.27 x 10^9^	-11.77	2.95	2.02	2.30	2.30
Melanoma	0.021	3.80 x 10^9^	2.48	199	7.64 x 10^11^	-19.94	0	0	0	0
Bone cancer (osteosarcoma)	0.00035^b^	4.18 x 10^6^	0.067	5	2.93 x 10^7^	-25.80	1.17	0.03	0.83	0.6
Thyroid cancer	0.011	6.50 x 10^7^	0.087	7	5.85 x 10^8^	-17.17	0.36	0.8	0.36	0.36

^a^sievert (Sv) is the weighted SI unit of radiation dose = 1 J kg^-1^ [18]

^b^based on Japanese atomic bomb survivors Life Span Study (LSS) cohort all leukemia mortality, Table 65 in UNSCEAR [14] Annex A

^c^taken from Tomasetti and Vogelstein [2];

^d^non-melanoma skin cancer incidence.

**Table 2:** Summary data on smoking-associated cancer risk and various tissue parameters, reproduced in part from Tomasetti & Vogelstein [2] and Doll *et al*. [15]

Cancer type	US lifetime natural cancer incidence [13]	Number of stem cells in tissue of origin	Numbers of divisions of each stem cell per year	Numbers of divisions of each stem cell over life	Number of divisions of all stem cell over lifetime	Extra Risk Score (ERS)	Mortality rate difference (/10^5^ / year) current vs former smokers [15]
Myeloid leukemia	0.007	1.35 x 10^8^	12	960	1.30 x 10^11^	-23.95	-1.3
Chronic lymphocytic leukemia	0.006	1.35 x 10^8^	12	960	1.30 x 10^11^	-24.69	1.8
Basal cell carcinoma	0.3^a^	5.82 x 10^9^	7.6	608	3.55 x 10^12^	-6.56	0
Colon cancer	0.045	2.00 x 10^8^	73	5840	1.17 x 10^12^	-16.25	-4.1
Esophageal cancer	0.005	8.64 x 10^5^	17.4	1390	1.20 x 10^9^	-20.89	14.3
Gallbladder cancer	0.003736^a^	1.60 x 10^6^	0.584	47	7.84 x 10^7^	-19.16	0.4
Brain cancer	0.006	1.35 x 10^8^	0.025	2	2.70 x 10^8^	-18.73	-6.2
Liver cancer	0.009	3.01 x 10^9^	0.9125	88	2.71 x 10^11^	-23.39	7.9
Trachea, bronchus and lung cancer	0.066	1.22 x 10^9^	0.07	5.6	9.27 x 10^9^	-11.77	180.2
Melanoma	0.021	3.80 x 10^9^	2.48	199	7.64 x 10^11^	-19.94	3.4
Pancreatic cancer	0.015	4.18 x 10^9^	1	80	3.43 x 10^11^	-21.04	8.9
Thyroid cancer	0.011	6.50 x 10^7^	0.087	7	5.85 x 10^8^	-17.17	-0.6

^a^taken from Tomasetti and Vogelstein [2];

**Table 3:** Linear regression fits of model (9) to data of Tomasetti and Vogelstein [2]^a^, and in some cases omitting tumors with short latency (leukemia, bone, thyroid).

Coefficients	Linear regression estimate (+95% CI)	*p*-value	R^2^
Full dataset			
*α*_1_ (coefficient of ln[*D* / *N*_0_])	0.524 (0.281, 0.767)	<0.001	0.646
*α*_2_ (coefficient of ln[*N*_0_])	0.540 (0.312, 0.769)	<0.001	
Analysis omitting tumors with short latency (leukemia, bone (osteosarcoma), thyroid)			
*α*_1_ (coefficient of ln[*D* / *N*_0_])	0.543 (0.207, 0.879)	0.003	0.473
*α*_2_ (coefficient of ln[*N*_0_])	0.485 (0.151, 0.818)	0.007	

^a^based on natural cancer risks, number of stem cells and cumulative stem-cell divisions in the second, fourth and seventh columns of Table S1 in Tomasetti and Vogelstein [2].

**Table 4:** Trends of linear regression model (11) fitted to radiation exposure-induced cancer incidence risk (REIC)(percent) (or log_10_[REIC]) with explanatory variables (a) numbers of stem-cell divisions per year (or log_10_[numbers of stem-cell divisions per year]), (b) log_10_[cumulative number of stem-cell divisions], (c) extra risk score (ERS) and (d) log_10_[number of stem-cells], using data of Tomasetti and Vogelstein [2], as in Table 1.

Independent variable and model	Cancer risk (REIC percent per Sv^a^ or log_10_[REIC percent per Sv^a^]) per unit of each independent variable (*α*_1_) (+95% CI)	*p*-value	Pearson / Spearman correlation coefficient	R^2^
REIC [Japan, BEIR VII weighted ERR/EAR model] vs number of stem-cell divisions per year	0.008 (-0.018, 0.033)	0.507	0.239 / -0.027	0.057
log_10_[REIC [Japan, BEIR VII weighted ERR/EAR model]] vs log_10_[number of stem-cell divisions per year]	0.033 (-0.240, 0.305)	0.778	0.119 / 0.143	0.014
REIC [Japan, ICRP weighted ERR/EAR model] vs number of stem-cell divisions per year	0.008 (-0.017, 0.034)	0.473	0.257 / 0.064	0.066
log_10_[REIC [Japan, ICRP weighted ERR/EAR model]] vs log_10_[number of stem-cell divisions per year]	0.044 (-0.231, 0.320)	0.707	0.159 / 0.262	0.025
REIC [Japan, ERR model] vs number of stem-cell divisions per year	0.005 (-0.028, 0.038)	0.740	0.120 / 0.070	0.015
log_10_[REIC [Japan, ERR model]] vs log_10_[number of stem-cell divisions per year]	0.010 (-0.296, 0.317)	0.938	0.033 / 0.262	0.001
REIC [Japan, EAR model] vs number of stem-cell divisions per year	0.009 (-0.015, 0.033)	0.388	0.307 / 0.088	0.094
log_10_[REIC [Japan, EAR model]] vs log_10_[number of stem-cell divisions per year]	0.085 (-0.396, 0.567)	0.680	0.174 / 0.310	0.030
REIC [Japan, BEIR VII weighted ERR/EAR model] vs log_10_[cumulative number of stem-cell divisions]	-0.011 (-0.345, 0.322)	0.939	-0.028 / -0.076	0.001
log_10_[REIC [Japan, BEIR VII weighted ERR/EAR model]] vs log_10_[cumulative number of stem-cell divisions]	0.051 (-0.151, 0.253)	0.560	0.244 / 0.214	0.060
REIC [Japan, ICRP weighted ERR/EAR model] vs log_10_[cumulative number of stem-cell divisions]	0.024 (-0.310,0.359)	0.870	0.060 / 0.076	0.004
log_10_[REIC [Japan, ICRP weighted ERR/EAR model]] vs log_10_[cumulative number of stem-cell divisions]	0.082 (-0.113, 0.277)	0.343	0.387 / 0.452	0.150
REIC [Japan, ERR model] vs log_10_[cumulative number of stem-cell divisions]	-0.068 (-0.485, 0.350)	0.717	-0.131 / -0.137	0.017
log_10_[REIC [Japan, ERR model]] vs log_10_[cumulative number of stem-cell divisions]	0.012 (-0.221, 0.244)	0.907	0.050 / 0.143	0.002
REIC [Japan, EAR model] vs log_10_[cumulative number of stem-cell divisions]	0.084 (-0.229, 0.397)	0.553	0.214 / 0.149	0.046
log_10_[REIC [Japan, EAR model]] vs log_10_[cumulative number of stem-cell divisions]	0.244 (-0.035, 0.523)	0.077	0.657 / 0.595	0.432
REIC [Japan, BEIR VII weighted ERR/EAR model] vs extra risk score	0.043 (-0.052, 0.138)	0.327	0.346 / 0.340	0.120
log_10_[REIC [Japan, BEIR VII weighted ERR/EAR model]] vs extra risk score	0.005 (-0.059, 0.068)	0.867	0.071 / 0.214	0.005
REIC [Japan, ICRP weighted ERR/EAR model] vs extra risk score	0.051 (-0.042, 0.144)	0.240	0.410 / 0.413	0.168
log_10_[REIC [Japan, ICRP weighted ERR/EAR model]] vs extra risk score	0.014 (-0.050, 0.077)	0.618	0.210 / 0.333	0.044
REIC [Japan, ERR model] vs extra risk score	0.044 (-0.079, 0.167)	0.432	0.281 / 0.207	0.079
log_10_[REIC [Japan, ERR model]] vs extra risk score	-0.006 (-0.078, 0.065)	0.833	-0.089 / 0.000	0.008
REIC [Japan, EAR model] vs extra risk score	0.069 (-0.010, 0.148)	0.079	0.580 / 0.681	0.336
log_10_[REIC [Japan, EAR model]] vs extra risk score	0.074 (-0.012, 0.161)	0.080	0.652 / 0.667	0.425
REIC [Japan, BEIR VII weighted ERR/EAR model] vs log_10_[number of stem cells]	0.299 (-0.421, 0.547)	0.773	0.105 / -0.006	0.011
log_10_[REIC [Japan, BEIR VII weighted ERR/EAR model]] vs log_10_[number of stem cells]	0.509 (-0.239, 0.364)	0.629	0.204 / 0.311	0.041
REIC [Japan, ICRP weighted ERR/EAR model] vs log_10_[number of stem cells]	0.110 (-0.370, 0.590)	0.612	0.184 / 0.117	0.034
log_10_[REIC [Japan, ICRP weighted ERR/EAR model]] vs log_10_[number of stem cells]	0.118 (-0.173, 0.408)	0.360	0.375 / 0.491	0.140
REIC [Japan, ERR model] vs log_10_[number of stem cells]	0.039 (-0.575, 0.652)	0.888	0.051 / -0.142	0.003
log_10_[REIC [Japan, ERR model]] vs log_10_[number of stem cells]	0.003 (-0.341, 0.347)	0.983	0.009 / 0.096	0.000
REIC [Japan, EAR model] vs log_10_[number of stem cells]	0.197 (-0.242, 0.636)	0.330	0.344 / 0.215	0.118
log_10_[REIC [Japan, EAR model]] vs log_10_[number of stem cells]	0.425 (0.078, 0.772)	0.024	0.775 / 0.659	0.600

^a^sievert (Sv) is the weighted SI unit of radiation dose = 1 J kg^-1^ [18]

**Table 5:** Trends of linear regression model (12) fitted to mortality rate difference [current smokers–former smokers] (/10^5^ /year) with explanatory variables (a) numbers of stem-cell divisions per year, (b) log_10_[cumulative number of stem-cell divisions], (c) extra risk score (ERS) and (d) log_10_[number of stem-cells], using data of Tomasetti and Vogelstein [2], as in Table 2.

Independent variable and model	Cancer risk (mortality rate difference [current–former smokers] / 10^5^ / year) per unit of each independent variable (*α*_1_) (+95% CI)	*p*-value	Pearson / Spearman correlation coefficient	R^2^
Smoking cancer risk vs number of stem-cell divisions per year	-0.469 (-2.212, 1.274)	0.562	-0.186 / -0.035	0.035
Smoking cancer risk vs log_10_[number of stem-cell divisions per year]	-17.715 (-49.015, 13.585)	0.236	-0.370 / -0.035	0.137
Smoking cancer risk vs log_10_[cumulative number of stem-cell divisions]	-3.095 (-25.972, 19.782)	0.769	-0.095 / 0.042	0.009
Smoking cancer risk vs extra risk score	3.741 (-2.702, 10.185)	0.225	0.379 / -0.182	0.143
Smoking cancer risk vs log_10_[number of stem cells]	6.957 (-21.246, 35.160)	0.595	0.171 / 0.169	0.029

**Table 6:** Trends of linear regression model (13) of log_10_[US lifetime natural cancer incidence risk] vs log_10_[cumulative stem-cell divisions]among cancer sites with available radiation risk (Table 1) or smoking risk (Table 2) data, and in the dataset of Tomasetti and Vogelstein [2], and in some cases omitting tumors with short latency (leukemia, bone, thyroid).

Natural cancer dataset used	log_10_[US lifetime natural cancer incidence risk] per unit of log_10_[cumulative stem-cell divisions] (*α*_1_) (+95% CI)	*p*-value	Pearson / Spearman correlation coefficient	R^2^
Full dataset				
Radiation data natural cancer risks (Table 1)^a^	0.349 (0.117, 0.580)	0.009	0.775 / 0.771	0.600
Smoking data natural cancer risks (Table 2)^b^	0.214 (0.017, 0.412)	0.036	0.608 / 0.732	0.369
Tomasetti and Vogelstein [2] data natural cancer risks ^c^	0.533 (0.383, 0.682)	<0.001	0.804 / 0.810	0.646
Analysis omitting tumors with short latency (leukemia, bone (osteosarcoma), thyroid)				
Radiation data natural cancer risks (Table 1)^a^	0.300 (-0.043, 0.643)	0.072	0.772 / 0.771	0.596
Smoking data natural cancer risks (Table 2)^b^	0.256 (0.031, 0.481)	0.031	0.713 / 0.817	0.509
Tomasetti and Vogelstein [2] data natural cancer risks ^c^	0.513 (0.260, 0.767)	<0.001	0.686 / 0.725	0.832

^a^based on natural cancer risks and cumulative stem-cell divisions in the second and sixth columns of Table 1;

^b^based on natural cancer risks and cumulative stem-cell divisions in the second and sixth columns of Table 2;

^c^based on natural cancer risks and cumulative stem-cell divisions in the second and seventh columns of Table S1 in Tomasetti and Vogelstein [2].

## Methods and Data

We tested the prediction of expression (6) via regression of the logarithm of the lifetime natural cancer risk, ln[*CR*], in relation to the log of the number of cell divisions per stem cell, ln[*D* / *N*_0_] and the log of the number of stem cells, ln[*N*_0_]. Specifically we fitted a model in which the log of the lifetime cancer risk is assumed to be given by:

$$\text{ln}[CR]=\alpha_{0}+\alpha_{1}\;\text{ln}[D/N_{0}]+\alpha_{2}\;\text{ln}[N_{0}]+\varepsilon$$ (9)

The data used is drawn from Tomasetti and Vogelstein [2], subject to minor alterations, relating to the entries for glioblastoma and medulloblastoma; the number of divisions per stem cell (over a lifetime) was derived by dividing the cumulative number of stem-cell divisions by the total number of stem cells. The number of divisions per stem cell per year was derived from this figure by dividing by 80 (the approximate mean lifetime, in years).

Tomasetti and Vogelstein [2] assess the extra-risk score (ERS), given by:

$$ERS={\text{ln}}_{10}[CR]{\text{ln}}_{10}[D]$$ (10)

It should be noted that *CR*, being a probability, is always less than or equal to 1, and *D* is usually substantially greater than 1, as shown in Tables 1 and 2, so that *ERS* will almost always be negative. Tomasetti and Vogelstein used an adjusted ERS measure, defined as *ERS* + 18.49; the figure 18.49 was derived from a *K*-means clustering analysis [2]. [Note: we shall retain the original definition of ERS given by Eq (10)–adding a constant makes no difference to any statistical inference using it.] They speculated that “if the ERS for a tissue type is high–that is, if there is a high cancer risk of that tissue type relative to its number of stem-cell divisions—then one would expect that environmental or inherited factors would play a relatively more important role in that cancer’s risk” [2]. For most of the analysis of Tables 4–6 we re-estimated *CR* for each cancer site, using baseline US cancer risks [13], rather than using the estimates given by Tomasetti and Vogelstein [2]; only for the analysis in the bottom row in Table 6 are the original cancer risks of Tomasetti and Vogelstein employed. For this reason the ERS for various endpoints is re-calculated using Eq (10), rather than using the values employed by Tomasetti and Vogelstein [2]. The re-estimated values *CR* and ERS are given in Tables 1 and 2.

A test can be made of the assumption of Tomasetti and Vogelstein [2] that ERS may be correlated with the variation in susceptibility of a tissue to environmental factors, using radiation-associated and smoking-associated cancer risk as examples of such factors, both mutagens that induce a large number of types of cancer [14, 15]. We considered radiation-exposure induced cancer incidence risk (REIC) evaluated by the United Nations Scientific Committee on the Effects of Atomic Radiation (UNSCEAR) [Table 70 in Annex A of [14]] for various cancer sites, as shown in Table 1; for leukemia we use radiation-exposure induced cancer death risk (REID) evaluated by UNSCEAR [Table 65 in Annex A of [14]]; mortality was used because leukemia incidence was not evaluated in the latest LSS cancer incidence report [16], a preliminary version of which formed the basis of the UNSCEAR evaluations [14]. This we do by fitting a model in which:

$$REIC=\alpha_{0}+\alpha_{1}ERS+\varepsilon$$ (11)

Similar models were fitted replacing the explanatory (independent) variable, *ERS*, by any of: (a) the cumulative number of stem-cell divisions over lifetime; (b) the number of stem-cell divisions per year; or (c) the number of stem cells, as we discuss below. From now on, in Eq (11) and subsequent discussions it is to be understood that REIC is replaced by REID whenever appropriate (i.e., for leukemia). We are most interested here in the parameter *α*_1_, the change in REIC per unit of *ERS*. The measures of REIC or REID are those estimated using a generalized absolute risk or generalized relative risk model fitted to current Japanese atomic bomb survivor data by UNSCEAR [14]. The cancer incidence [16] and mortality [17] data derived from the Japanese atomic bomb survivor Life Span Study (LSS) cohort are the primary basis for most sets of risk estimates obtained by national and international radiation safety committees, such as the International Commission on Radiological Protection (ICRP) [18], UNSCEAR [14], and the Biological Effects of Ionizing Radiation (BEIR) committee [19]. There is considerable uncertainty as to how one should transfer radiation risk estimates between populations. We have used lifetime population risk estimates for a current population that is as close as possible to the LSS cohort, namely the Japanese population. However, even in this case it is not clear how one transfers risk from the wartime-exposed LSS cohort, subject to a variety of privations, to a current Japanese population [20]. The two most common methods of transfer are based on risk models expressed in terms of the excess relative risk (ERR) or the excess absolute risk (EAR) produced by exposure to ionizing radiation during the atomic bombings. The use of the terms ERR and EAR in this context is slightly misleading, as most scientific committees [18, 19] have developed models of relative and absolute excess risk with adjustment for attained age, age at exposure and other risk-modifying variables. Risk transfer is often assumed to be intermediate between additive (i.e., based on EAR models) or multiplicative (i.e., based on ERR models) [20], and ICRP [18] and BEIR [19] have developed independent cancer-site-specific weighting schemes for the contributions made by the EAR and ERR models. For this reason, we have used both REIC based on both of these (ICRP [18], BEIR [19]) schemes in Table 4, as well as those based on pure EAR or ERR transfer.

Likewise, we assess the correlations of smoking-associated cancer risk using data on differences in mortality rates between current and former smokers, *Sm*_*diff*_, in the British doctors’ cohort [15], as shown in Table 2, fitting a model in which:

$$Sm_{diff}=\alpha_{0}+\alpha_{1}ERS+\varepsilon$$ (12)

We are most interested here in the parameter *α*_1_, the change in *Sm*_*diff*_ per unit of *ERS*. We tested the correlations of site-specific cancer risk with:

1. number of stem-cell divisions per year (or log_10_[number of stem-cell divisions per year]);
2. log_10_[cumulative number of stem-cell divisions over lifetime];
3. ERS; and
4. log_10_[total number of stem cells].

Tests for association were performed by fitting linear models with dependent measures:

1. REIC (percent Sv^-1^) (or log_10_[REIC]); and
2. difference between mortality rate (per 10^5^ per year) for current smokers and former smokers in the British doctors’ cohort [15].

Finally, we judged it important to assess the effect of selection of cancer sites involved in selecting radiation-associated and tobacco-associated risk. Tables 1 and 2 represent a selected subset of the 31 cancer sites considered by Tomasetti and Vogelstein [2], namely those with data on radiation or smoking risk, numbering 10 and 12 endpoints respectively. As such it is possible that we may have inadvertently selected cancers that have a different pattern of risk. To test this possibility, we fitted a linear regression model of log_10_[lifetime cancer incidence risk], in relation to log_10_[cumulative stem-cell divisions] to these two subsets, as well as the original Tomasetti and Vogelstein data, via the model:

$$\text{ln}[CR]=\alpha_{0}+\alpha_{1}\;\text{ln}[D]+\varepsilon$$ (13)

We are most interested here in the parameter *α*_1_, the change in ln[*CR*] per unit of ln[*D*].

Because of the possibility of heterogeneity in the types of tumor being considered, and in particular differences between tumors in the number of driver mutations, for certain sensitivity analyses (Tables 3 and 6 and Tables A and B in S1 Text) we evaluated model fits restricting to those common epithelial sites that were judged to have a rather larger number of critical driver mutations, specifically omitting three cancer types (leukemia, bone cancer (osteosarcoma), thyroid cancer) that appear to have a shorter latency of induction after radiation exposure [14].

All linear regressions were performed via ordinary least squares [21], using R [22]. The *p*-values shown in Tables 4–6 were generally estimated using an *F*-test [21], and are in relation to the respective trend parameters (*α*_1_, *α*_2_). In Table 3 the *p*-values for each parameter (*α*_1_, *α*_2_) were estimated using a 2-sided *t*-test [21]. The data on stem cell turnover used (Tables 1 and 2) are largely taken from Tomasetti and Vogelstein [2] (with minor modifications for glioblastoma as indicated above). However, for the purposes of fitting models (11) and (12) we employed slightly different estimates of lifetime cancer risk [13], and correspondingly modified estimates of ERS, derived via expression (10).

## Results

Table 3 details the fit of model (6), and indicates that the power of *D* / *N*_0_ (adjusted for *N*_0_) is *α*_1_ = 0.524 (95% CI 0.281, 0.767), and the power of *N*_0_ (adjusted for *D* / *N*_0_) is *α*_2_ = 0.540 (95% CI 0.312, 0.769), i.e., both *α*_1_ and *α*_2_ are considerably less than 1. The Table shows that the analyses are essentially unchanged if those tumors with short latency (leukemia, bone, thyroid) are omitted from the analysis.

Tables 4 and 5 and Figures A and B in S4 Text show that there is no evidence of associations (*p*>0.05) of any of these measures (number of stem-cell divisions per year, log_10_[cumulative number of stem-cell divisions over lifetime], ERS, log_10_[number of stem cells]) with REIC or smoking mortality rate difference, regardless of the model used. There are borderline-significant increasing trends of various measures of radiation risk, specifically log_10_[REIC] with log_10_[cumulative number of stem-cell divisions] (*p* = 0.077), REIC with ERS (*p* = 0.079) and log_10_[REIC] with log_10_[number of stem cells] (*p* = 0.024) (Table 4), and these models are also the only ones with R^2^>0.2, although generally weaker trends (*p*>0.2) and smaller R^2^ (<0.2) are observed with risks estimated using the BEIR VII or ICRP weighted EAR/ERR models. The analyses are essentially unchanged if those tumors with short latency (leukemia, bone, thyroid) are omitted from the analysis (Tables A and B in S1 Text), although no trends approach statistical significance (*p*>0.1) and most R^2^ are small (all but four are <0.2).

The results of fitting model (13), reported in Table 6, suggests that there are significant (*p* = 0.009) trends of log_10_[lifetime natural cancer incidence risk] vs unit of log_10_[cumulative stem-cell divisions] in the set of 10 radiation-cancer sites (Table 1), as also (*p* = 0.036) in the set of 12 tobacco-cancer sites (Table 2). Table 6 also shows that the analyses are essentially unchanged if those tumors with short latency (leukemia, bone, thyroid) are omitted from the analysis.

## Discussion

As outlined in the Introduction, one would expect cancer rate to be proportional to the *k*th power of the expected number of cell divisions per stem cell, with *k* in the range 5–7 as implied by the shape of the age-incidence relationship for most cancers [3]. By expression (6) this implies that the cancer rate should be proportional to a power of the expected number of divisions per stem cell, in other words proportional to ${\left[D/N_{0}\right]}^{F((g_{i}),k)}$ with *F*((*g*_*i*_),*k*) between 3.08 and 3.45. We have shown that the cancer rate is proportional to a power of the expected number of divisions per stem cell with 95% upper CI that is less than 0.8 (Table 3), about a quarter of the lower limit of the suggested range, 3.08, implying some inconsistency with the age-incidence relationship and the predictions of a multistage carcinogenesis model, if one makes the strong assumption of homogeneity of numbers of driver mutations across cancer sites, which we discuss below. Analysis of ERS and various other measures considered by Tomasetti and Vogelstein suggests that these are poorly predictive of radiation- or smoking-associated cancer risk (Tables 4 and 5). There is weak evidence at a marginal levels of statistical significance (*p* = 0.024–0.080) of trend for four measures, two in relation to ERS, one in relation to cumulative stem-cell divisions, and one in relation to number of stem cells (Table 4). However, the probability of four or more independent events out of the 37 tested trends in Tables 4 and 5, each with probability *p*, is $1-{\left(1-p\right)}^{37}-37p{\left(1-p\right)}^{36}-\frac{37\cdot 36}{1\cdot 2}p^{2}{\left(1-p\right)}^{35}-\frac{37\cdot 36\cdot 35}{1\cdot 2\cdot 3}p^{3}{\left(1-p\right)}^{34}$, which takes the value 0.218 when the mean *p*-value of these four, *p* = 0.065, is substituted. If the 37 non-significant (*p*>0.1) trends in Tables A and B in S1 Text are included in this total then the analogous calculation is $1-{\left(1-p\right)}^{74}-74p{\left(1-p\right)}^{73}-\frac{74\cdot 73}{1\cdot 2}p^{2}{\left(1-p\right)}^{72}-\frac{74\cdot 73\cdot 72}{1\cdot 2\cdot 3}p^{3}{\left(1-p\right)}^{71}$, which takes the value 0.716 when the mean *p*-value of *p* = 0.065 is substituted. These results do not therefore suggest anything other than chance findings.

Tomasetti and Vogelstein performed additional analysis, correlating lifetime number of stem cell divisions or total number of stem cells with data on the EAR or ERR at exposure age 30, taken from Table 11 of Preston *et al*. [16], described in a somewhat summarial way in an online technical report [23]. Tomasetti and Vogelstein [23] observed no correlation between lifetime number of stem cell divisions, or total number of stem cells, and radiation-associated EAR or ERR, to some extent paralleling our findings. Tomasetti and Vogelstein argue that this absence of correlation implies that “the correlation … found between cancer risk and total number of stem cell divisions is not due to the effects of environmental factors, but rather to replicative mutations” [23]. Their later analysis takes no account of the fact that there is not a single number that describes radiation-associated ERR or EAR, which for most cancer sites are strongly modified by age at exposure, and attained age, as is clear in any case from Preston *et al*. [16], and from much other data [14]. Using only a single number for ERR or EAR, for exposure age 30 for each cancer site is therefore somewhat arbitrary, and does not adequately take account of the lifetime radiation-associated cancer risk, which we judge to be the more legitimate quantity; in the present paper we have assessed these radiation-associated risks in a number of ways, via REIC and REID. There has been no parallel analysis of smoking data by Tomasetti and Vogelstein [23], so that in any case most of the analysis we have done, reported in Tables 3–5 and Tables A and B in S1 Text, looking not simply at lifetime number of stem cell divisions or numbers of stem cells, but also number of stem-cell divisions per year and logarithmic transformations of these measures, is not paralleled in this online report [23].

It might be inferred from the use that Tomasetti and Vogelstein [2] make of logarithmically transformed variables (e.g., lifetime cancer risk, total stem cell divisions) in their analysis that relative risk would be the most relevant measure with which to assess “extrinsic” environmentally-driven and “intrinsic” cell-replication driven risk. However, as is clear from Table 4 there is little difference made whether one uses relative risk models, absolute risk models or some mixture of the two for evaluating lifetime radiation risk.

A notable recent paper of Wu *et al*. [24] reanalyzed the data of Tomasetti and Vogelstein [2]. Their analysis, combined with insights gained from a mathematical cancer model that they developed, suggested that “intrinsic [non-replicative] factors contribute only modestly (less than ~10–30% of lifetime risk) to cancer development”, a strikingly different assessment from that made by Tomasetti and Vogelstein [2].

A critical assumption in our analysis of the power-age relationship is that the underlying process is describable by the multistage model of Armitage and Doll, with a constant number of driver mutation stages *k*. This implies that the incidence at age *t* a power of age is approximately *CN*_0_*t*^*k*−1^ [4, 8, 11]. While this is the case for most cancers considered here, with a range of the exponent *k* between 5 and 7 [3], it is certainly not the case for certain pediatric tumors, in particular acute lymphocytic leukemia, which is not one of the tumors considered by Tomasetti and Vogelstein [2]. One possible way in which our analysis might underestimate the slope of the relationship between ln[cancer risk] and ln[stem-cell divisions per stem cell] (i.e. ln[*D* / *N*_0_]), which by the expression (6) corresponds to *F*((*g*_*i*_),*k*) (a monotonic increasing function of *k*), would be if there were a strong negative correlation between the number of driver mutations and the number of stem cell divisions by cancer type. This would not be expected–if anything one would expect that the correlation should go in the opposite direction. The organism cannot afford to have tissues where there is a high rate of stem cell divisions, but where a single mutation can lead to cancer. This is supported by the data–indeed the tumors with short latency (leukemia, bone (osteosarcoma), thyroid), and presumably therefore having a smaller number of cancer driver mutations than the remaining (and rather larger) group of epithelial tumors, tend to have numbers of stem-cell mutations per year, or in total, that are less than the mean, and for bone and thyroid cancer are among the smallest values in aggregate (Tables 1 and 2). We have also performed sensitivity analyses excluding the tumors with short latency (leukemia, bone (osteosarcoma), thyroid); the results of this analysis are essentially the same as the main analysis (Tables 3 and 6 and Tables A and B in S1 Text), suggesting that material bias would not result from such heterogeneity.

It is an inevitable weakness of the analysis that we conduct that we combine across cancer types. We have just single measurements of cell turnover per cancer endpoint, and likewise single estimates of the various cancer risk measures per endpoint. Nevertheless, to the extent that the model of Armitage and Doll, with the generalization we employ to take account of intermediate compartment growth rates [10, 12], discussed in the Introduction, provides a unifying framework, with a constant number of driver mutation stages *k*, as discussed above, this may be legitimate.

In recent years there has been movement away from use of the Armitage and Doll model [4], which postulates a series of independent rate-limiting mutations and in the form commonly used also makes use of an approximation to the conditional likelihood, to stochastically “exact” models that allow for intermediate cell proliferation or apoptosis. Examples of this alternative approach include the two-mutation model of Moolgavkar, Venzon, and Knudson [25, 26], and various generalizations of this that allow for a larger number of mutational stages [27], and the incorporation of various types of genomic instability [28, 29]. US population colon cancer incidence can be described by such a model incorporating just two rate-limiting mutations, combined with a destabilizing mutational event [29–31]. Nevertheless the Armitage-Doll model, or slight modifications thereof, is still much used. For example, Tomasetti *et al*. [10] used a modified Armitage-Doll model with adjustment for clonal expansion of cells with various numbers of driver mutations fitted to epidemiological data combined with genome-wide sequencing data to infer that three rate-limiting mutations adequately describe lung and colorectal cancer incidence. We employed the same modified Armitage-Doll model here.

A detailed justification of the ERS measure proposed by Tomasetti and Vogelstein [2] is not provided in their paper. Tomasetti and Vogelstein [2] state that “the greater the absolute value of this product is, the smaller is the evidence for the presence of any environmental or inherited factor acting on that tissue.” They give a numerical example to suggest why this product may perform better in this respect than the corresponding ratio, ln_10_[*CR*] / ln_10_[*D*], but this is the only justification provided. Tomasetti and Vogelstein motivate derivation of the ERS, as determining “when there is high cancer risk of that tissue type relative to its number of stem cell divisions” [2]. As noted by Potter and Prentice the ERS is calculated “not as the ratio, but as the product, of cancer incidence rates and stem cell division number”, meaning that “the resulting classification into D [deterministic] and R [replicative] tumors does not seem interpretable” [32].

In practice, the score produces odd results. For example melanoma, acute myeloid leukemia and esophageal cancer all have negative scores, putatively suggestive of predominantly stochastic effects, but there are known strong environmental influences on all three [33]. As we have shown, there is little evidence to suggest that this measure, or various other plausible measures based on cell-turnover data assembled by Tomasetti and Vogelstein [2] are quantitatively associated with the susceptibility of a tissue to radiation- or smoking-associated cancer. Moreover, the slope of the relationship between log[cancer incidence rate] and log[expected number of stem-cell divisions] is much less than 1 (Table 6), and therefore much less than the value of 2–3, or even more, that would be expected from either the Armitage and Doll model [3, 4] or other carcinogenesis models [25, 28–30], as discussed above in relation to the analysis of Table 3.

A problem with our analysis is that we only have quantitative information on radiation-associated and tobacco-associated cancer risk for a subset of the 31 natural cancer sites considered by Tomasetti and Vogelstein, where radiation or smoking risk data are available. While the significance of the increasing trends of log_10_[lifetime natural cancer incidence risk] *vs* unit of log_10_[cumulative stem-cell divisions] (i.e., *α*_1_) is preserved in the sets of radiation- and tobacco-cancer data that we consider, the trends are of somewhat reduced magnitude compared with those in the natural cancer dataset of Tomasetti and Vogelstein [2] (Table 6). Their paper [2] has been criticized by O’Callaghan for the somewhat heterogeneous biological data, of rather variable quality [34]. The biological data also came from a number of different populations, and so it may be problematic comparing it with natural cancer incidence risks for a US population. This is therefore also a problem in our analysis, largely based as it is on the Tomasetti and Vogelstein stem-cell turnover data, which we compare with Japanese population radiation risks, or British tobacco risks. Because the radiation- or tobacco-associated risks are not available for the specific cancer subtypes cited by Tomasetti and Vogelstein [2], we use aggregate (e.g., whole lung, esophagus, brain) radiation- or tobacco-associated risks, and comparable US lifetime cancer incidence risks [13]. Tobacco smoke is for most smokers a largely continuous exposure, in contrast to the instantaneous dose of radiation received by the Japanese atomic bomb survivors; this may have some bearing on the slightly stronger trends we observe for radiation risk. It is possible that some of the variation in smoking-associated risk is related to the degree to which tissues are exposed to the range of carcinogens in tobacco smoke. Thus high risks are found for lung, larynx, esophagus, oropharyngeal, bladder, stomach, kidney, and cervical cancer, less so for most other organs [35]. It is possible that some part of the differences in tissue exposure may overwhelm differences driven by number of cell divisions.

Based on the results of their analysis, Tomasetti and Vogelstein “suggest that only a third of the variation in cancer risk among tissues is attributable to environmental factors or inherited predispositions. The majority is due to “bad luck,” that is, random mutations arising during DNA replication in normal, noncancerous stem cells.” [2] However, it is known that stem-cell division may be caused by external influences, as for example during tissue recovery from cytotoxic agents [36]. Even if it were true that random errors during stem-cell division are involved in the etiology of a great majority of cancers, it would be incorrect to infer that most cancers are just due to “bad luck” and do not involve environmental or lifestyle factors. This is a simple consequence of the multistage nature of carcinogenesis, which implies that multiple mutational events contribute to a given cancer. For example, strong skepticism was expressed when, based on extrapolations from epidemiological studies of underground miners, it was projected that about 10–20% of all lung cancers in the U.S. population might be due to radon [37, 38]. This seemed unrealistic, given the evidence that smoking accounted for 90% of all lung cancers [35]. However, when it is recognized that the great majority of radon-induced lung cancers also involved smoking, the problem disappears. Indeed even if, say, half of all mutations were due to random errors during stem cell division, the requirement for 3–7 mutations to produce a cancer would imply that the great majority (>85%) of all cancers could involve one or more non-random mutational event. Related arguments have been made by Song and Giovannucci [39]. Epidemiological analysis suggests that in excess of 40% of cancers in the US are caused by exogenous exposures, the dominant cause being tobacco [40]. Regardless of any multiple somatic mutation scenario, it is probably unwise to judge the importance of factors by thinking of them as “residues” after other factors are somehow subtracted out. To judge whether a factor is important, the most reasonable approach to inference on the importance of a factor in relation to some cancer endpoint is to use data where that factor is present.

In summary, the data used by Tomasetti and Vogelstein as the basis of their assertion that “the incorporation of a replicative component as a … quantitative determinant of cancer risk forces rethinking of our notions of cancer causation” [2] is in conflict with predictions of a theoretical multistage model of carcinogenesis. Their statement that “if the ERS for a tissue type is high … then one would expect that environmental … factors would play a relatively more important role in that cancer’s risk” [2] is in conflict with the lack of correlation between ERS and other stem-cell proliferation indices and radiation- or smoking-related cancer risk.

## Supporting Information

S1 Text Supplementary Analysis. Tables A, B, Analogs of Tables 4 and 5, omitting tumors with short latency (leukemia, thyroid, bone).
(DOCX)

S2 Text Datasets used for analysis. (ZIP)

S3 Text R code and output files used for analysis. (ZIP)

S4 Text Supplementary Figures A, B. (DOCX)
