# Supplementary material for: Lack of Correlation between Stem-Cell Proliferation and Radiation- or Smoking-Associated Cancer Risk
Source: PLoS One. 2016 Mar 31;11(3):e0150335. doi: 10.1371/journal.pone.0150335 (PMC4816383; doi:10.1371/journal.pone.0150335)
Supplement: S1 Text — Tables A, B, Analogs of Tables 4 and 5, omitting tumors with short latency (leukemia, thyroid, bone). (DOCX) [file pone.0150335.s001.docx]

**S1 Text. Supplementary Analysis. Tables A, B, Analogs of Tables 4 and 5, omitting tumors with short latency (leukemia, thyroid, bone).**

**Table A. Analog of Table 4 in the main paper, omitting tumors with short latency (leukemia, thyroid, bone (osteosarcoma)). Trends of linear regression model (11) fitted to radiation exposure-induced cancer incidence risk (REIC)(percent) (or log_10_[REIC]) with explanatory variables (a) numbers of stem-cell divisions per year (or log_10_[numbers of stem-cell divisions per year]), (b) log_10_[cumulative number of stem-cell divisions], (c) extra risk score (ERS) and (d) log_10_[number of stem-cells], using data of Tomasetti and Vogelstein [2], as in Table 1 of main paper.**

| Independent variable and model | Cancer risk (REIC percent per Sv^a^ or log_10_[REIC percent per Sv^a^]) per unit of each independent variable ($\alpha_{1}$ ) (+95% CI) | *p*-value | Pearson / Spearman correlation coefficient | R^2^ |
| --- | --- | --- | --- | --- |
| REIC [Japan, BEIR VII weighted ERR/EAR model] vs number of stem-cell divisions per year | 0.008 (-0.035, 0.050) | 0.649 | 0.238 / 0.257 | 0.057 |
| log_10_[REIC [Japan, BEIR VII weighted ERR/EAR model]] vs log_10_[number of stem-cell divisions per year] | 0.034 (-0.541, 0.608) | 0.864 | 0.107 / 0.300 | 0.012 |
| REIC [Japan, ICRP weighted ERR/EAR model] vs number of stem-cell divisions per year | 0.008 (-0.035, 0.050) | 0.650 | 0.237 / 0.257 | 0.056 |
| log_10_[REIC [Japan, ICRP weighted ERR/EAR model]] vs log_10_[number of stem-cell divisions per year] | 0.026 (-0.563, 0.615) | 0.897 | 0.081 / 0.300 | 0.007 |
| REIC [Japan, ERR model] vs number of stem-cell divisions per year | 0.005 (-0.050, 0.060) | 0.820 | 0.121 / 0.314 | 0.015 |
| log_10_[REIC [Japan, ERR model]] vs log_10_[number of stem-cell divisions per year] | 0.022 (-0.611, 0.655) | 0.919 | 0.064 / 0.400 | 0.004 |
| REIC [Japan, EAR model] vs number of stem-cell divisions per year | 0.009 (-0.030, 0.047) | 0.564 | 0.299 / 0.029 | 0.090 |
| log_10_[REIC [Japan, EAR model]] vs log_10_[number of stem-cell divisions per year] | -0.027 (-0.818, 0.765) | 0.922 | -0.061 / 0.000 | 0.004 |
| REIC [Japan, BEIR VII weighted ERR/EAR model] vs log_10_[cumulative number of stem-cell divisions] | 0.002 (-0.716, 0.719) | 0.995 | 0.003 / 0/314 | 0.000 |
| log_10_[REIC [Japan, BEIR VII weighted ERR/EAR model]] vs log_10_[cumulative number of stem-cell divisions] | 0.095 (-0.359, 0.550) | 0.552 | 0.360 / 0.600 | 0.129 |
| REIC [Japan, ICRP weighted ERR/EAR model] vs log_10_[cumulative number of stem-cell divisions] | 0.018 (-0.702, 0.738) | 0.948 | 0.035 / 0.314 | 0.001 |
| log_10_[REIC [Japan, ICRP weighted ERR/EAR model]] vs log_10_[cumulative number of stem-cell divisions] | 0.120 (-0.328, 0.567) | 0.457 | 0.441 / 0.600 | 0.194 |
| REIC [Japan, ERR model] vs log_10_[cumulative number of stem-cell divisions] | -0.053 (-0.957, 0.850) | 0.877 | -0.082 / 0.086 | 0.007 |
| log_10_[REIC [Japan, ERR model]] vs log_10_[cumulative number of stem-cell divisions] | 0.054 (-0.471, 0.579) | 0.764 | 0.186 / 0.300 | 0.035 |
| REIC [Japan, EAR model] vs log_10_[cumulative number of stem-cell divisions] | 0.072 (-0.581, 0.725) | 0.774 | 0.152 / 0.257 | 0.023 |
| log_10_[REIC [Japan, EAR model]] vs log_10_[cumulative number of stem-cell divisions] | 0.212 (-0.331, 0.755) | 0.302 | 0.583 / 0.500 | 0.340 |
| REIC [Japan, BEIR VII weighted ERR/EAR model] vs extra risk score | 0.060 (-0.148, 0.268) | 0.469 | 0.371 / 0.657 | 0.137 |
| log_10_[REIC [Japan, BEIR VII weighted ERR/EAR model]] vs extra risk score | 0.024 (-0.124, 0.172) | 0.645 | 0.283 / 0.600 | 0.080 |
| REIC [Japan, ICRP weighted ERR/EAR model] vs extra risk score | 0.067 (-0.140, 0.272) | 0.423 | 0.407 / 0.657 | 0.166 |
| log_10_[REIC [Japan, ICRP weighted ERR/EAR model]] vs extra risk score | 0.034 (-0.111, 0.179) | 0.511 | 0.394 / 0.600 | 0.155 |
| REIC [Japan, ERR model] vs extra risk score | 0.067 (-0.200, 0.335) | 0.523 | 0.330 / 0.371 | 0.109 |
| log_10_[REIC [Japan, ERR model]] vs extra risk score | 0.011 (-0.157, 0.179) | 0.849 | 0.119 / 0.200 | 0.014 |
| REIC [Japan, EAR model] vs extra risk score | 0.074 (-0.104, 0.253) | 0.313 | 0.500 / 0.771 | 0.250 |
| log_10_[REIC [Japan, EAR model]] vs extra risk score | 0.072 (-0.093, 0.237) | 0.261 | 0.624 / 0.700 | 0.389 |
| REIC [Japan, BEIR VII weighted ERR/EAR model] vs log_10_[number of stem cells] | 0.082 (-0.796, 0.960) | 0.807 | 0.129 / 0.086 | 0.017 |
| log_10_[REIC [Japan, BEIR VII weighted ERR/EAR model]] vs log_10_[number of stem cells] | 0.103 (-0.479, 0.685) | 0.613 | 0.309 / 0.600 | 0.095 |
| REIC [Japan, ICRP weighted ERR/EAR model] vs log_10_[number of stem cells] | 0.109 (-0.767, 0.985) | 0.747 | 0.170 / 0.086 | 0.029 |
| log_10_[REIC [Japan, ICRP weighted ERR/EAR model]] vs log_10_[number of stem cells] | 0.150 (-0.413, 0.712) | 0.460 | 0.439 / 0.600 | 0.193 |
| REIC [Japan, ERR model] vs log_10_[number of stem cells] | 0.083 (-1.029, 1.195) | 0.845 | 0.104 / -0.200 | 0.011 |
| log_10_[REIC [Japan, ERR model]] vs log_10_[number of stem cells] | 0.050 (-0.616, 0.715) | 0.828 | 0.136 / 0.200 | 0.018 |
| REIC [Japan, EAR model] vs log_10_[number of stem cells] | 0.158 (-0.627, 0.943) | 0.606 | 0.269 / 0.143 | 0.073 |
| log_10_[REIC [Japan, EAR model]] vs log_10_[number of stem cells] | 0.354 (-0.178, 0.886) | 0.125 | 0.774 / 0.700 | 0.599 |

^a^sievert (Sv) is the weighted SI unit of radiation dose = 1 J kg^-1^ [[18](#_ENREF_18)]

**Table B. Analog of Table 5 in the main paper, omitting tumors with short latency (leukemia, thyroid, bone (osteosarcoma)). Trends of linear regression model (12) fitted to mortality rate difference [current smokers – former smokers] (/10^5^ /year) with explanatory variables (a) numbers of stem-cell divisions per year, (b) log_10_[cumulative number of stem-cell divisions], (c) extra risk score (ERS) and (d) log_10_[number of stem-cells], using data of Tomasetti and Vogelstein [2], as in Table 2 of main paper, omitting tumors with short latency (leukemia, thyroid, bone (osteosarcoma)).**

| Independent variable and model | Cancer risk (mortality rate difference [current – former smokers] / 10^5^ / year) per unit of each independent variable ($\alpha_{1}$ ) (+95% CI) | *p*-value | Pearson / Spearman correlation coefficient | R^2^ |
| --- | --- | --- | --- | --- |
| Smoking cancer risk vs number of stem-cell divisions per year | -0.522 (-2.707, 1.663) | 0.590 | -0.209 / -0.033 | 0.044 |
| Smoking cancer risk vs log_10_[number of stem-cell divisions per year] | -22.465 (-66.305, 21.374) | 0.265 | -0.416 / -0.033 | 0.173 |
| Smoking cancer risk vs log_10_[cumulative number of stem-cell divisions] | -4.083 (-34.598, 26.433) | 0.761 | -0.119 / -0.150 | 0.014 |
| Smoking cancer risk vs extra risk score | 4.061 (-5.315, 13.438) | 0.340 | 0.361 / -0.367 | 0.130 |
| Smoking cancer risk vs log_10_[number of stem cells] | 5.865 (-29.825, 41.555) | 0.709 | 0.145 / 0.017 | 0.021 |
