# Supplementary figures and images for: Lack of Correlation between Stem-Cell Proliferation and Radiation- or Smoking-Associated Cancer Risk
Source: PLoS One. 2016 Mar 31;11(3):e0150335. doi: 10.1371/journal.pone.0150335 (PMC4816383; doi:10.1371/journal.pone.0150335)

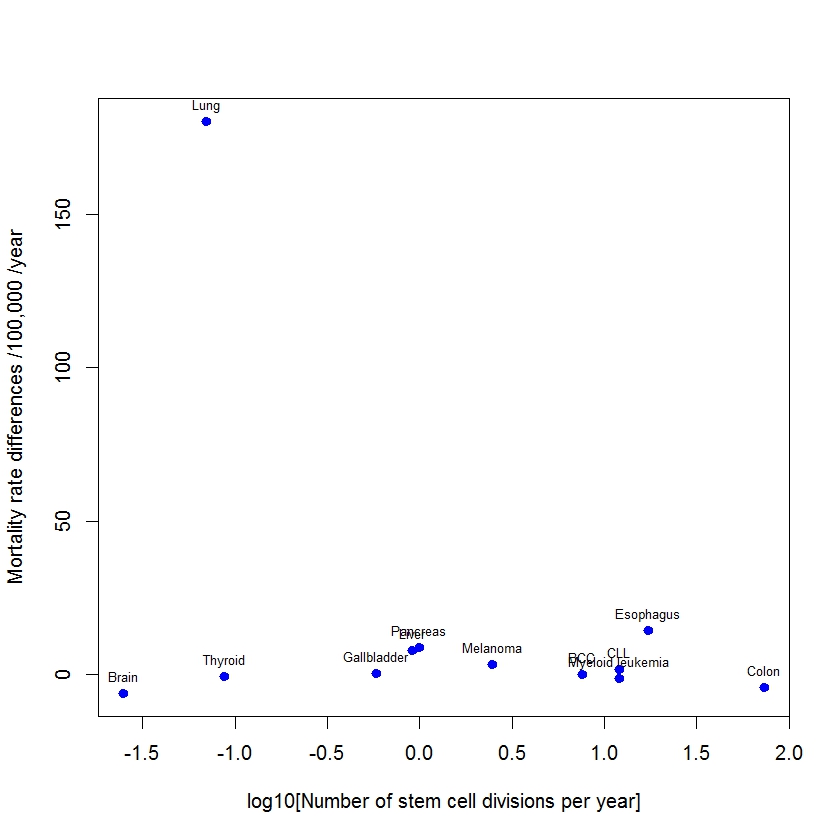

Supplement: S3 Text — (ZIP) [file pone.0150335.s003.zip › Smoking difference vs log(divisions per year).jpeg]

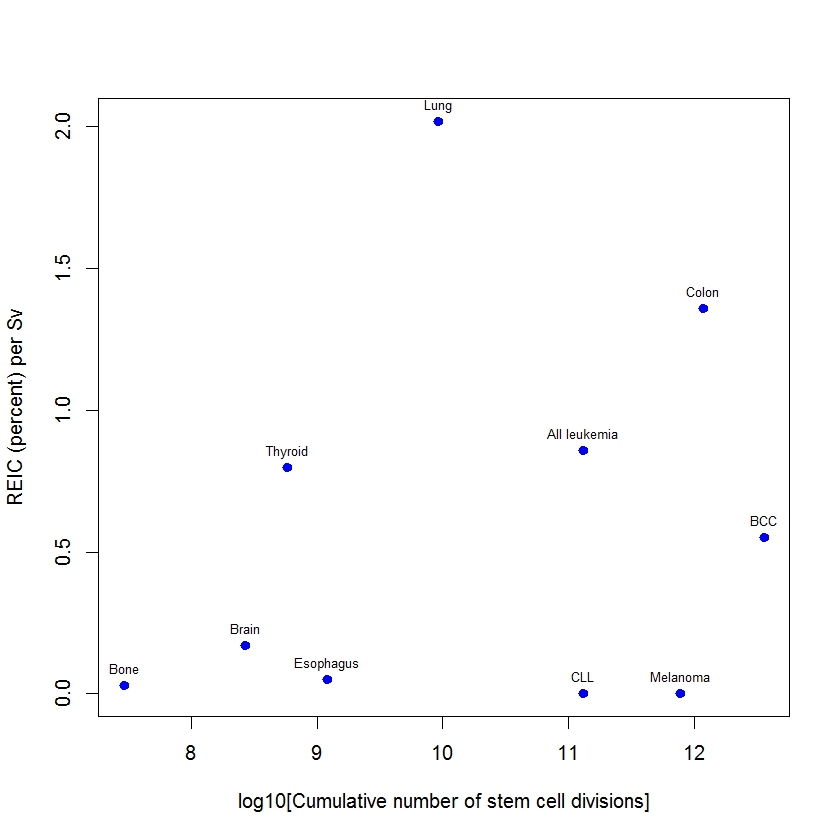

Supplement: S3 Text — (ZIP) [file pone.0150335.s003.zip › REIC vs cumulative divisions.jpeg]

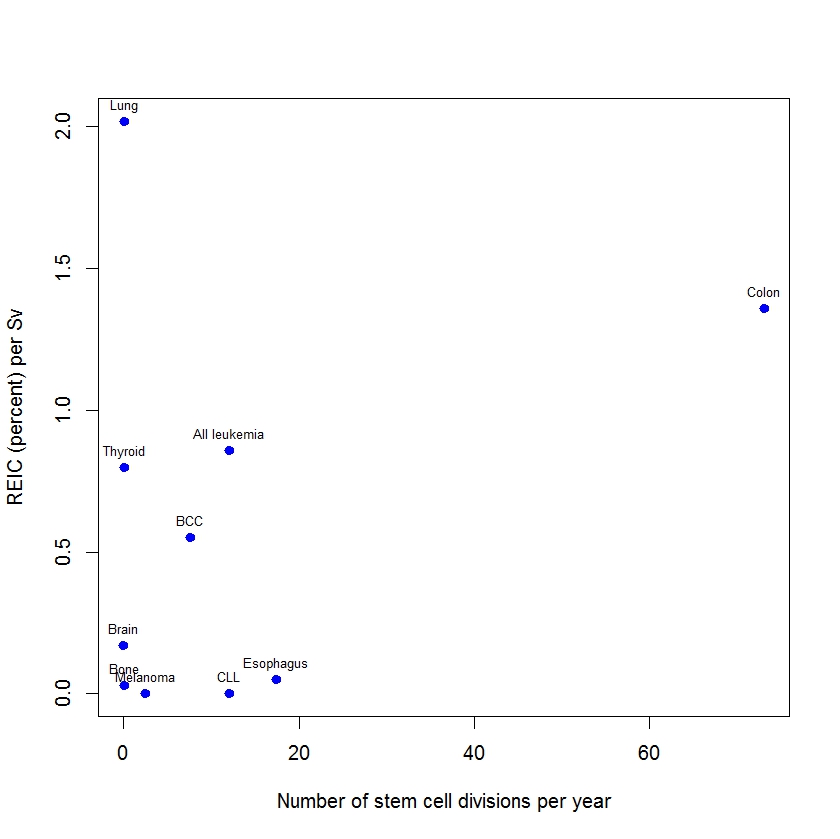

Supplement: S3 Text — (ZIP) [file pone.0150335.s003.zip › REIC vs divisions per year.jpeg]

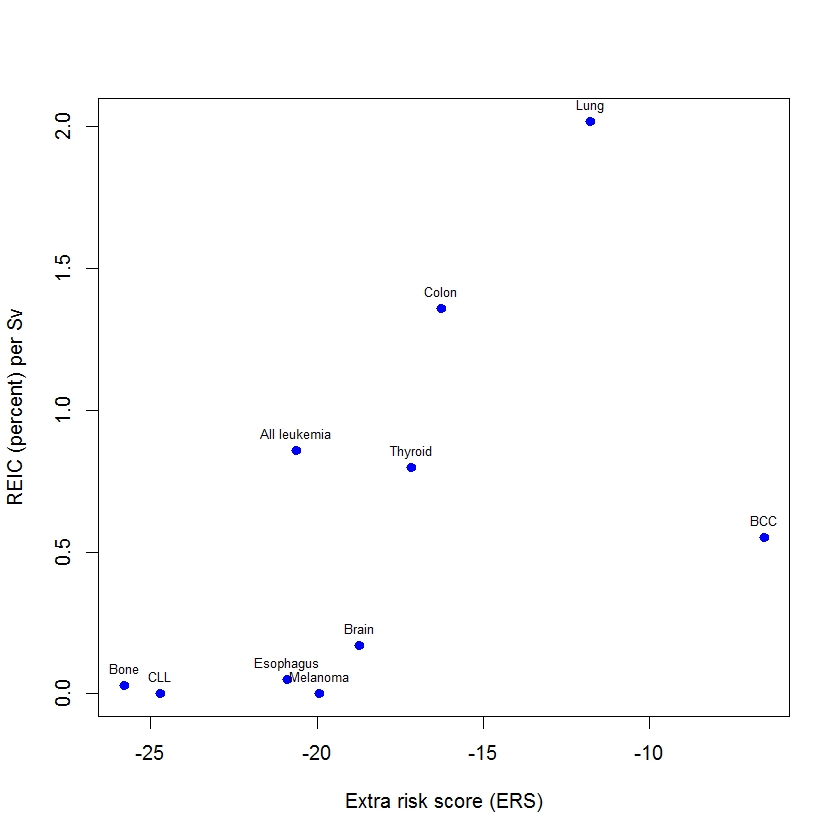

Supplement: S3 Text — (ZIP) [file pone.0150335.s003.zip › REIC vs ERS.jpeg]

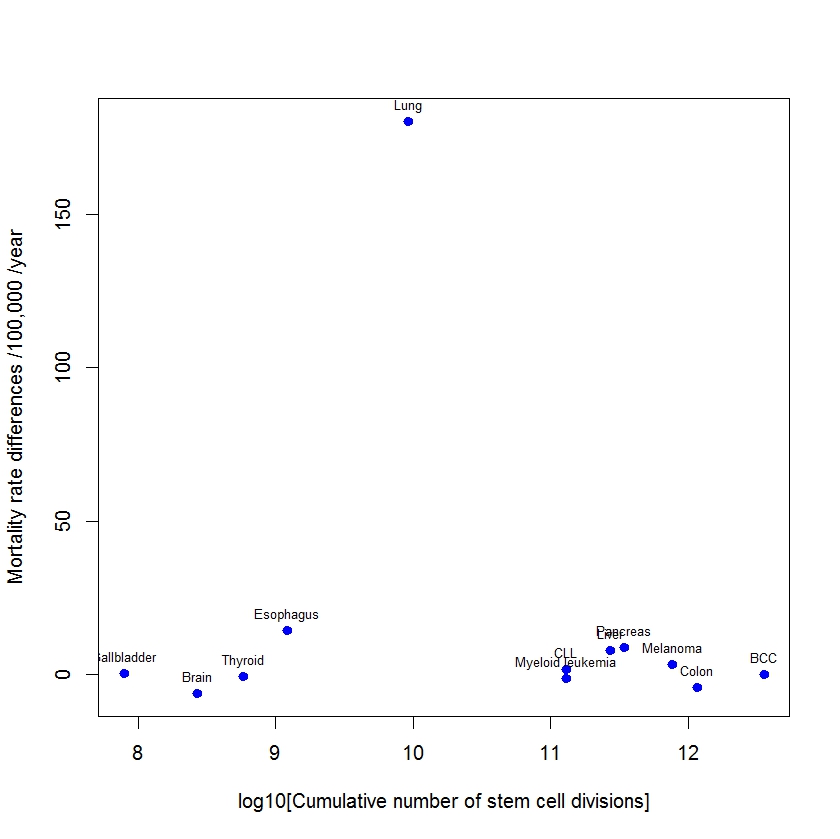

Supplement: S3 Text — (ZIP) [file pone.0150335.s003.zip › Smoking difference vs cumulative divisions.jpeg]

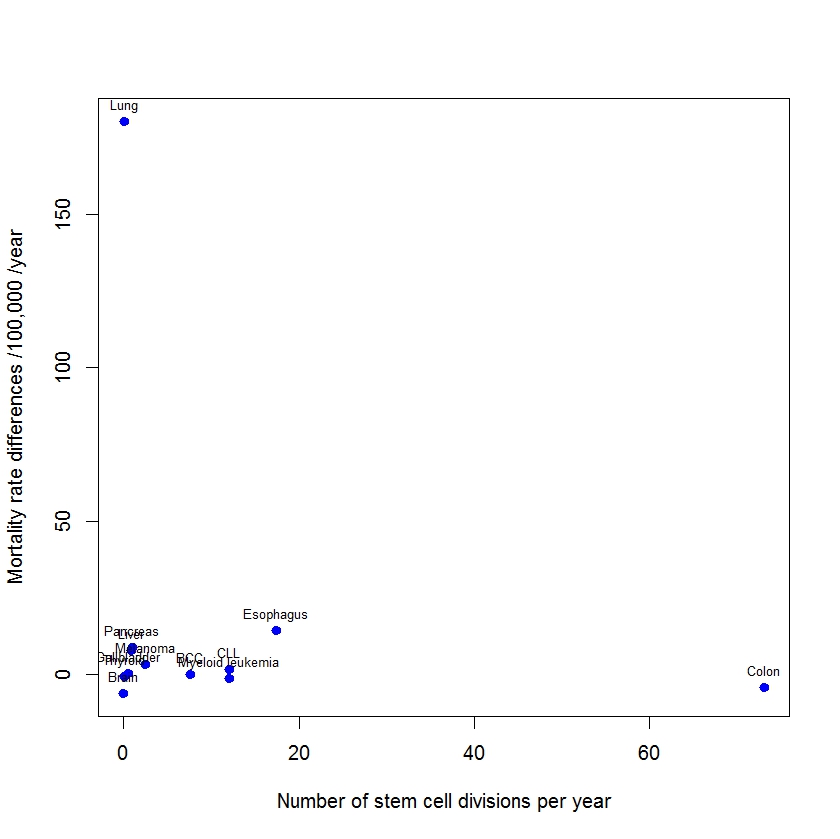

Supplement: S3 Text — (ZIP) [file pone.0150335.s003.zip › Smoking difference vs divisions per year.jpeg]

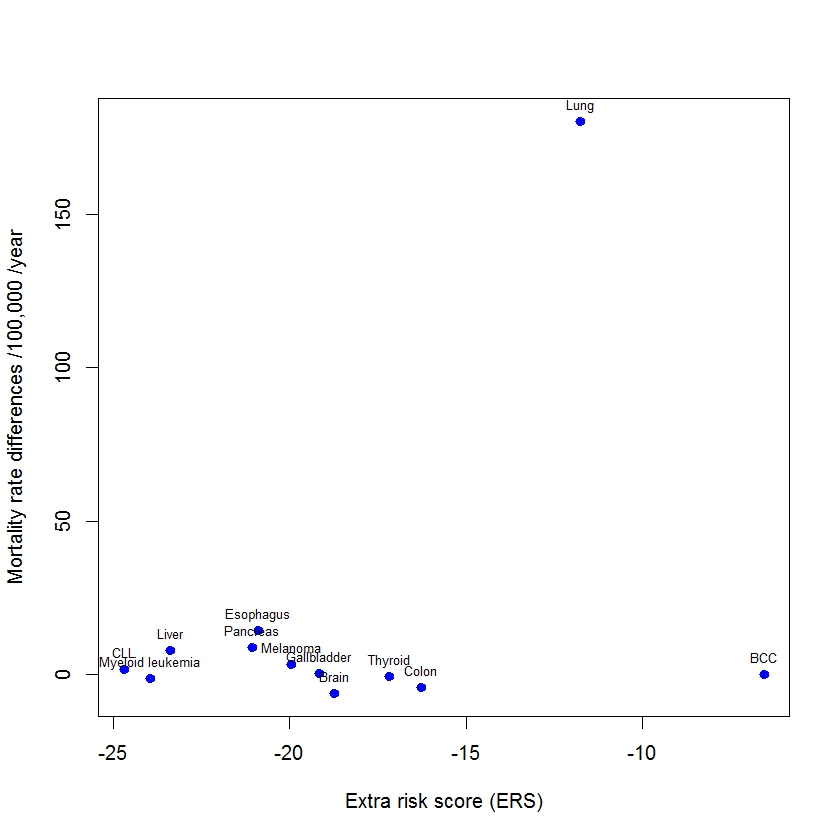

Supplement: S3 Text — (ZIP) [file pone.0150335.s003.zip › Smoking difference vs ERS.jpeg]
