# Supplementary material for: Lack of Correlation between Stem-Cell Proliferation and Radiation- or Smoking-Associated Cancer Risk
Source: PLoS One. 2016 Mar 31;11(3):e0150335. doi: 10.1371/journal.pone.0150335 (PMC4816383; doi:10.1371/journal.pone.0150335)
Supplement: S4 Text — (DOCX) [file pone.0150335.s004.docx]

**S4 Text. Supplementary Figures A, B.**

**Figure Legends**

Figure A. Trends of radiation exposure-induced cancer incidence risk (REIC)(percent), using excess absolute risk model for a Japanese population, with numbers of stem-cell divisions per year, log_10_[cumulative number of stem-cell divisions], and extra-risk score (ERS).

Figure B. Trends of mortality rate difference (current vs former smokers) (/10^5^ /year) with numbers of stem-cell divisions per year, log_10_[cumulative number of stem-cell divisions], and extra-risk score (ERS).

**Figure A.**

| numbers of stem-cell divisions per year |
| --- |
| 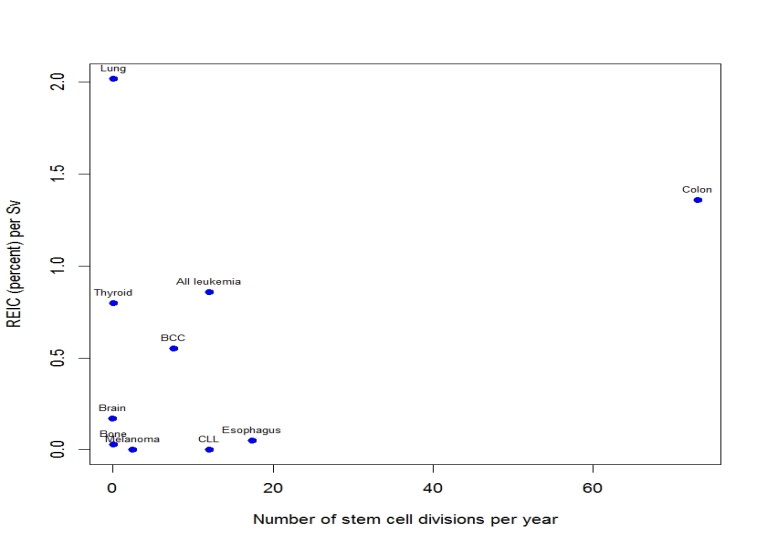 |
| log_10_[cumulative number of stem-cell divisions] |
| 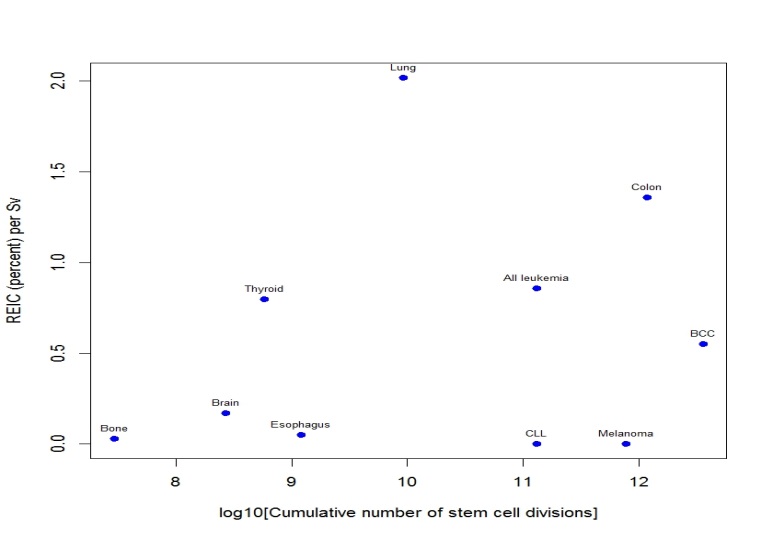 |
| extra risk score |
| 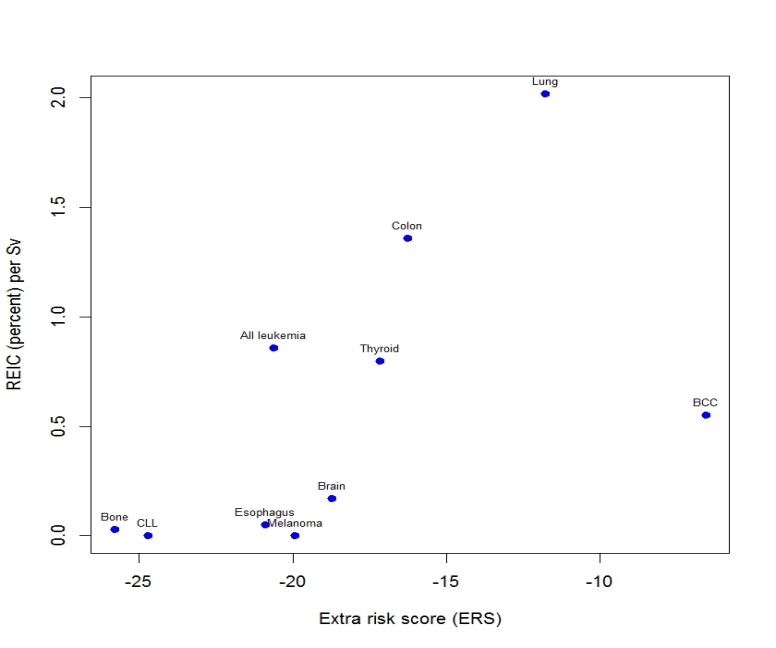 |

**Figure B.**

| numbers of stem-cell divisions per year |
| --- |
| 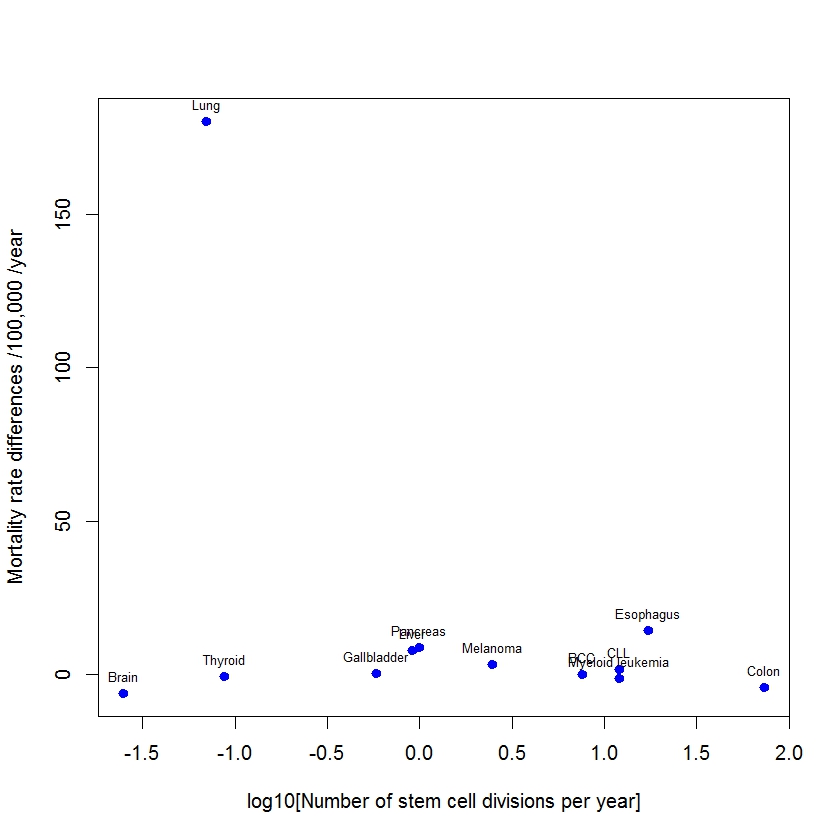 |
| log_10_[cumulative number of stem-cell divisions] |
| 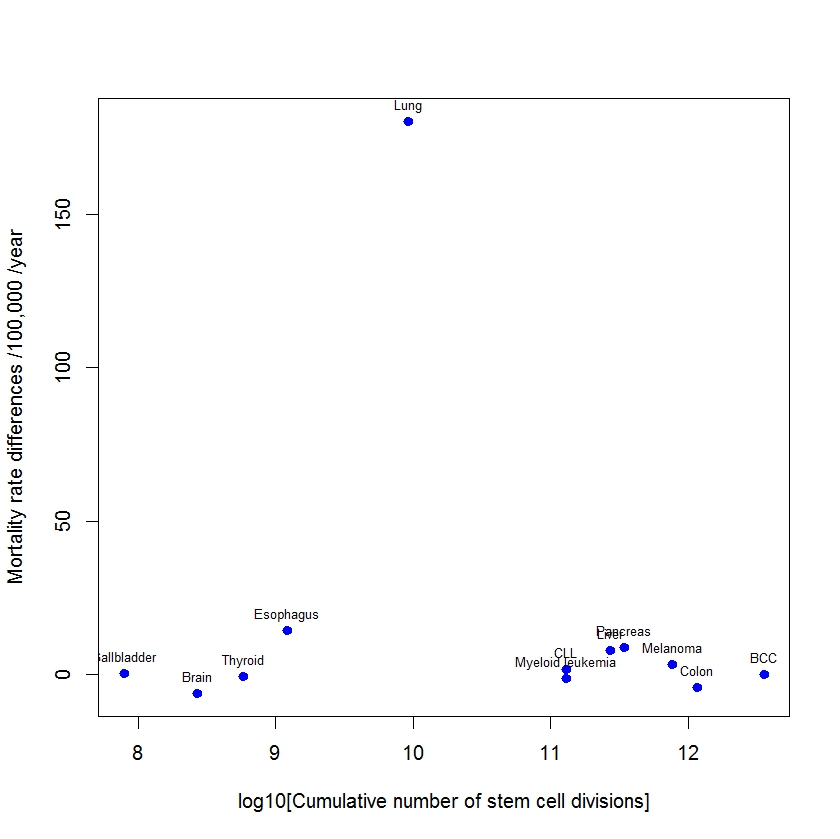 |
| extra risk score |
| 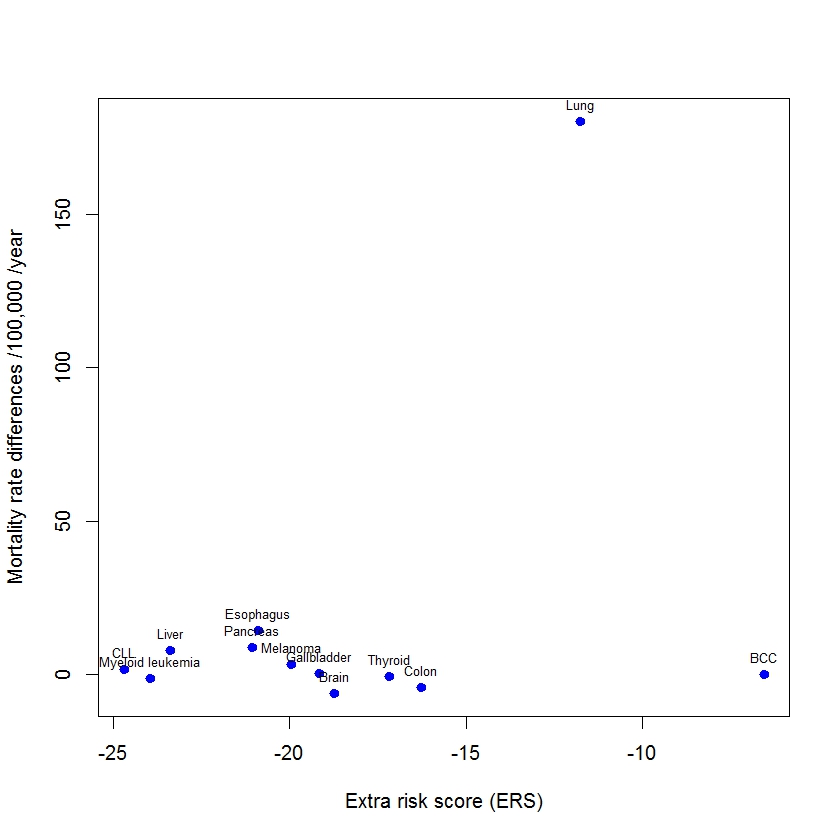 |
